# Author's Reply

**DOI:** 10.1371/journal.pmed.0020086

**Published:** 2005-03-29

**Authors:** Margaret Gatz

**Affiliations:** University of Southern CaliforniaLos Angeles, CaliforniaUnited States of America

Grant [1] describes as incorrect the statement that at least half of the explanation for individual differences in risk for Alzheimer disease is genetic. He suggests instead that dietary and lifestyle factors explain the majority of individual susceptibility to Alzheimer disease.

The basis for asserting a 50% or greater role for genetics in Alzheimer disease risk comes from family studies and from twin studies. In family studies, first-degree relatives of individuals with Alzheimer disease are at more than double the risk of Alzheimer disease compared to those with no affected relatives [2,3]. In twin studies, across different Scandinavian twin registries, estimates of heritability of Alzheimer disease range from 55% to over 70% [4].

Genetic risk undoubtedly represents the cumulative influence of many genes, including apolipoprotein E (APOE) and other genes not yet identified. In particular, it appears that the magnitude of the genetic component of Alzheimer disease risk is similar across ethnic communities, but that different genetic factors may contribute differently to that risk in white, Latino, and African American families [5].

Further, there are interactions between genetic and environmental risks, for example, between the APOE e4 allele and high cholesterol [6] or head injury [7].

Clearly Alzheimer disease is the outcome of multiple genetic and multiple environmental influences, operating additively and interactively. If genetic effects account for half of individual differences in liability, then environmental influences also account for half of the variation in susceptibility. From a public health viewpoint, it is vital to identify those influences that are modifiable. Controlling blood pressure and avoiding head trauma are examples. However, it is also important to appreciate that individuals bring differences in genetic risk to the table.
